# Annotations

**Published:** 1896-08-22

**Authors:** 


### ANNOTATIONS.

### The Vaccination Report.

The report of the Royal Commission on Vaccination, after finding that vaccination diminishes the liability to be attacked by small-pox, modifies the character of this disease and renders it less fatal, goes on to consider how best to encourage the use of this method of prophylaxis. In regard to this the Commissioners to some extent cut their coat according to their cloth, and, being convinced that no proposal to substitute for the pecuniary penalty any more stringent penalty, such as imprisonment, would have any chance of acceptance, they make various other suggestions the leading idea, underlying several of which is that while a parent who has conscientious objections to vaccination should, after undergoing certain formalties, be excused submitting their children to the process, these formalities should be of such a nature, and should involve such an amount of trouble as to prevent parents who are merely negligent from allowing their children to grow up unvaccinated on the pretence of conscientious scruples. It no doubt has been felt that certain parents have a grievance in the matter, and that the making of martyrs tend in some degree to elevate this grievance into a cult. If, however, anyone could avoid vaccination by attending before a local authority and making a statutory declaration of the reasons for his objection, the grievance and its advertisement would cease, while if the formalities were somewhat troublesome, those who at present let their children remain unvaccinated merely from fashion or from negligence would have them vaccinated as the least irksome alternative. It is also suggested that every duly qualified medical man who vaccinates a child successfully might be entitled to the same fee which is now paid to the public vaccinator, but with the important proviso that all children so vaccinated should be liable to inspection, and that the fee should not be paid unless the results were satisfactory. There can be no doubt that of recent years a very large amount of unsatisfactory vaccination has been performed, and it is to be hoped that some such plan as is here proposed, according to which private practice vaccination will be subject to inspection, will be of service in removing what is one of the main causes of the disrepute which has fallen on the operation in the public eye. Various other recommendations are made, to which we shall refer on a future occasion.

### Coroners' Juries and Hospitals.

"Serious allegations against hospital authorities" is the heading of a newspaper paragraph which records the death from accident of Mr. William Edward Read, of Basingstoke. Mr. Read, who, his sister admitted in evidence, occasionally got the worse for drink, had been visiting friends in London, and was found by a policeman lying on the footway at Waterloo Bridge, with a deep cut on the back of his head, after midnight on Friday last. He was taken to St. Thomas's Hospital, where the wound was attended to, and he was then sent away. The house surgeon saw definitely that he had been drinking, and that his accident was not serious. Later, Mr. Read, on attempting to cross the road in the direction of Waterloo Station, appears to have been knocked down by a passing railway van, the wheels of which ran over him. He died five hours later, and, according to the evidence of the house surgeon on night duty, from the shock of the second accident. The coroner's jury took it upon themselves to censure the hospital authorities for not keeping Mr. Read all night when he was taken to the hospital after the first accident, and Mr. Schroeder, the coroner, concurred in that view. Now, the truth is that hospital authorities have a rule to the effect that drunken cases, although they may be taken in and their immediate necessities attended to, cannot be retained in bed until they are sober. Manifestly hospitals are not refuges for people going home intoxicated, even though they may be the victims of accidents, unless, of course, a particular accident is obviously likely to be fatal. But Mr. Read's first accident was not serious, and his fall under the wheels of the van was no doubt due to his intoxicated condition. We are quite sure that hospital authorities are as humane as coroners' juries—even as coroners. Moreover, there is no doubt that they know their business quite as well. For a coroner's jury, therefore, to add such a rider to their verdict as the Lambeth jury did, and for the coroner to endorse it, was a piece of very foolish conduct, to say the least. It may sometimes happen in the administration of hospital rules that real harm is done. In Mr. Read's case it does not appear to thave been so. But even though occasional mishaps arise from strict obedience to orders, such large hospitals as those in London find strict rules absolutely indispensable, and strict enforcement equally indispensable. On no other principle could such institutions be carried on at all.

### The Advance of Dentistry.

The British Dental Association brought to a close last Friday a very successful congress. It is now beginning to be admitted that dentistry is a *bonâ fide* medical, or rather surgical speciality, as indeed, what else can it be? Dentistry, in fact, is one of the most indispensable of all the specialisms with which medicine some time ago threatened to be swamped. We are glad to see that Mr. Christopher Heath, Dr. Glover, Mr. Butlin, and some other eminent members of the medical profession, held out the hand of cordial fellowship to the dentists on the occasion of their meeting. It was not, perhaps, to be wondered at that the dental profession for some years was looked coldly upon by the medical. Many of its members were ill-educated, or rather quite uneducated; and many practices were tolerated, and some yet are not extinct, which were hardly consistent with the dignity of a learned profession. But the dentists who are worthy of the name are making resolute efforts to change all that. We are glad to see that they are anxious to have one or more direct representatives on the General Medical Council. This we hold to be both legitimate and laudable, and we hope their wishes will be cordially met in the matter. When a profession, by its most representative members, does its utmost to elevate itself in everything that is worthy, it ought to receive, more especially at the hands of kindred professions, all possible encouragement and help.

